# EyeLoop: An Open-Source System for High-Speed, Closed-Loop Eye-Tracking

**DOI:** 10.3389/fncel.2021.779628

**Published:** 2021-12-09

**Authors:** Simon Arvin, Rune Nguyen Rasmussen, Keisuke Yonehara

**Affiliations:** ^1^Department of Biomedicine, Danish Research Institute of Translational Neuroscience - DANDRITE, Nordic-EMBL Partnership for Molecular Medicine, Aarhus University, Aarhus, Denmark; ^2^Multiscale Sensory Structure Laboratory, National Institute of Genetics, Mishima, Japan; ^3^Department of Genetics, The Graduate University for Advanced Studies (SOKENDAI), Mishima, Japan

**Keywords:** oculographic tools, eye movement, eye movement abnormalities, software, Python (programming language), closed loop

## Abstract

Eye-trackers are widely used to study nervous system dynamics and neuropathology. Despite this broad utility, eye-tracking remains expensive, hardware-intensive, and proprietary, limiting its use to high-resource facilities. It also does not easily allow for real-time analysis and closed-loop design to link eye movements to neural activity. To address these issues, we developed an open-source eye-tracker – EyeLoop – that uses a highly efficient vectorized pupil detection method to provide uninterrupted tracking and fast online analysis with high accuracy on par with popular eye tracking modules, such as DeepLabCut. This Python-based software easily integrates custom functions using code modules, tracks a multitude of eyes, including in rodents, humans, and non-human primates, and operates at more than 1,000 frames per second on consumer-grade hardware. In this paper, we demonstrate EyeLoop’s utility in an open-loop experiment and in biomedical disease identification, two common applications of eye-tracking. With a remarkably low cost and minimum setup steps, EyeLoop makes high-speed eye-tracking widely accessible.

## Introduction

At every moment, the brain uses its senses to produce increasingly complex features that describe its external world (Ehinger et al., 2015; Zeki, 2015). Our everyday behaviors, such as navigating in traffic, are directed in large part by our sensory input, that is, what we see, hear, feel, etc. (Lee, 1980). The eyes, in particular, engage in sensory facilitation; For example, the optomotor response of insects detects perturbations of visual flow to avoid collisions (Theobald et al., 2010) and elicits stabilizing head movements in mice (Kretschmer et al., 2017). Similarly, combined eye-head movements in free-roaming mice were recently shown to re-align the visual axis to the ground plane, suggesting that vision itself is subject to sensory modulation (Meyer et al., 2020). Tracking the state of the eyes is thus often integral to nervous systems research.

Eye-tracking is widely used in neuroscience, from studying brain dynamics to investigating neuropathology and disease models (Yonehara et al., 2016; Wang et al., 2018; Meyer et al., 2020). Despite this broad utility, commercial eye-tracking systems, such as ISCAN (de Jeu and De Zeeuw, 2012; Yaramothu et al., 2018), remain expensive, hardware-intensive, and proprietary, constraining use to high-resource facilities. Likewise, deep learning-based approaches, such as *DeepLabCut* (Nath et al., 2019), still require specialized processing units and, for the most part, are limited to offline tracking. More generally, current systems tend to be programmatically rigid, e.g., by being compiled into executable, proprietary software unavailable for modifications, or coded in a more complex syntax and system architecture with advanced software modules. To address these issues, we developed an open-source eye-tracker – EyeLoop – tailored to investigating visual dynamics at very high speeds. EyeLoop enables low-resource facilities access to eye-tracking and encourages community-based development of software code through a modular, tractable algorithm based on high-level Python 3.

## Materials and Methods

### Ethics Statement

All experiments on mice were performed according to standard ethical guidelines and were approved by the Danish National Animal Experiment Committee (2020-15-0201-00452). No experiment on non-human primate was conducted in this study. The video footage of human eyes was provided by a human volunteer. The video footage of marmoset eyes was provided by Jude Mitchell (University of Rochester).

### Experimental Animals

Wild-type control mice (C57BL/6J) were obtained from Janvier Labs. *Frmd7*™ mice are homozygous female or hemizygous male *Frmd7*^*tm*1*b(KOMP)Wtsi*^ mice, which were obtained as *Frmd7*^*tm*1*a(KOMP)Wtsi*^ from the Knockout Mouse Project (KOMP) Repository, Exon 4 and neo cassette flanked by loxP sequences were removed by crossing with female Cre-deleter *Edil3*^*Tg(Sox*2–*cre)*1*Amc*/J^ mice (Jackson laboratory stock 4,783) as confirmed by PCR of genome DNA and maintained in a C57BL/6J background. Experiments were performed on 3 male and female wild-type control mice, and 3 male and female *Frmd7*™ mice. All mice were between 2 and 4 months old. Mice were group-housed and maintained in a 12 h/12 h light/dark cycle with *ad libitum* access to food and water.

### Head-Plate Implantation

Surgeries and preparation of animals for experiments were performed as previously described (Rasmussen et al., 2020). Mice were anesthetized with an intraperitoneal injection of fentanyl (0.05 mg/kg body weight; Hameln), midazolam (5.0 mg/kg body weight; Hameln), and medetomidine (0.5 mg/kg body weight; Domitor, Orion) mixture dissolved in saline. The depth of anesthesia was monitored by the pinch withdrawal reflex throughout the surgery. Core body temperature was monitored using a rectal probe and temperature maintained at 37-38°C by a feedback-controlled heating pad (ATC2000, World Precision Instruments). Eyes were protected from dehydration during the surgery with eye ointment (Oculotect Augengel). The scalp overlaying the longitudinal fissure was removed, and a custom head-fixing head-plate was mounted on the skull with cyanoacrylate-based glue (Super Glue Precision, Loctite) and dental cement (Jet Denture Repair Powder) to allow for subsequent head fixation during video-oculographic tracking. Mice were returned to their home cage after anesthesia was reversed with an intraperitoneal injection of flumazenil (0.5 mg/kg body weight; Hameln) and atipamezole (2.5 mg/kg body weight; Antisedan, Orion Pharma) mixture dissolved in saline, and after recovering on a heating pad for 1 h.

### Visual Stimulation

Visual stimulation was generated and presented *via* Python-based custom-made software (as EyeLoop *Extractor* modules). The visual stimulus was presented on a “V”-shaped dual-monitor setup (monitor size 47.65 × 26.87 cm, width x height) positioned 15 centimeters from the eye at an angle of 30° from the midline of the mouse. Each display thus subtended 115.61° in azimuth and 80.95° in elevation. This setup was adapted from a previous study (Rasmussen et al., 2021), which enabled us to cover most of the visual field of the mouse to evoke consistent visual responses. To evoke the optokinetic reflex in *Frmd7* knockout and wild-type mice, we presented a square-wave drifting grating simulating binocular rotation. Drifting gratings were presented in eight trials for 30 s at a time with 4 s of the gray screen between presentations and were drifted in two different directions along the horizontal axis (0° and 180°; monocular and binocular; parallel and anti-parallel) with a spatial frequency of 0.05 cycles/° and a speed of 5°/s.

### Rodent Video-Oculography

The mouse was placed on a platform with its head fixed to prevent head motion interference. Head fixation was achieved using a metallic plate implanted cranially. To minimize obstruction of the visual field-of-view, a 45° hot mirror was aligned above the camera and lateral to the rodent. The camera was positioned below the field-of-view due to space constraints in our experimental setup. Two PC monitors were positioned as described in subsection *Visual Stimulation*. Behind the right monitor, a near-infrared light source was angled at 45°. A CCD camera (Allied Vision Guppy Pro F-031 1/4″ CCD Monochrome Camera) was connected to the PC *via* a dedicated frame grabber PCIe expansion card (ADLINK FIW62). Using an EyeLoop *Importer*, *vimba.py* for Vimba-based cameras, the camera frames were fed to EyeLoop in real-time (fixed at ∼120 Hz). Finally, the standard EyeLoop data acquisition module continuously logged the generated tracking data.

### Software Availability

The software described here – EyeLoop – is freely available online, see https://github.com/simonarvin/eyeloop. For extensive sample data and information, see https://github.com/simonarvin/eyeloop_playground.

### Principles of EyeLoop

**FIGURE 1:**
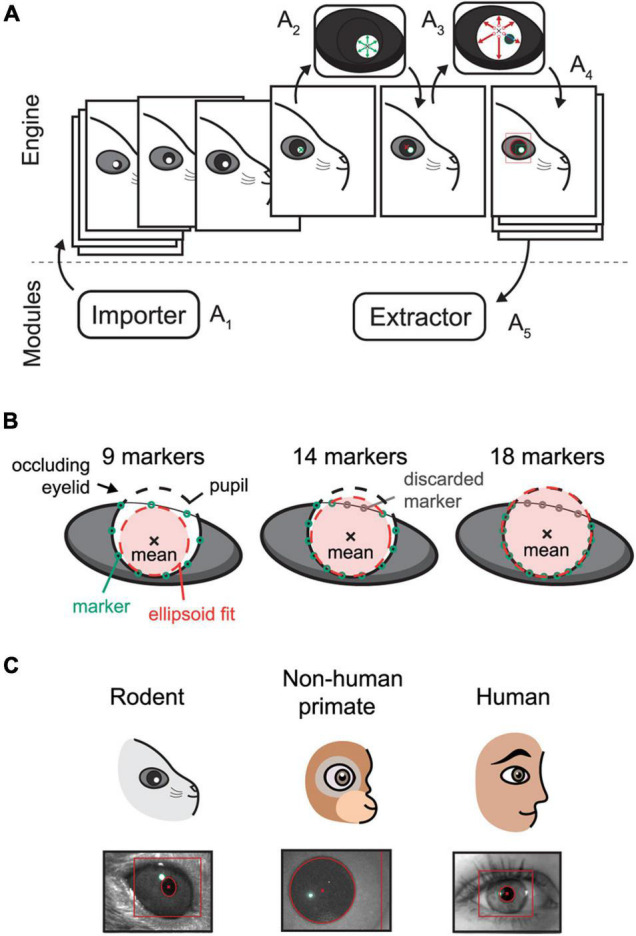
Schematic overview of the EyeLoop algorithm and its applications. **(A)** Software overview. The engine exchanges data with the modules. The Importer module imports camera frames in a compatible format **(A_1_)**. The frame is binarized and the corneal reflections are detected by a walk-out algorithm **(A_2_)**. Using the corneal reflections, any pupillary overlap is removed, and the pupil is detected *via* walk-out **(A_3_)**. Finally, the data is formatted in JSON and passed to all modules, such as for rendering **(A_4_)**, or data acquisition and experiments **(A_5_)**. **(B)** Occlusion filtering. EyeLoop tracks 32 points along the pupil contour by default. By computing the statistical mean of the data points and the standard deviation difference to the mean, EyeLoop filters the points to discard “bad” markers. Users can increase the number of markers in-code to produce better fits. **(C)** EyeLoop accepts a variety of animal eyes, including rodents, non-human primates, and humans, by employing distinct mathematical models for each type of eye.

EyeLoop is based on the versatile programming language, Python 3 (Python Software Foundation), using no proprietary software modules. Contrary to other frameworks used for eye-tracking, such as LabView (Sakatani and Isa, 2004), MATLAB (Cornelissen et al., 2002), or ISCAN (de Jeu and De Zeeuw, 2012; Yaramothu et al., 2018), Python is open-source software and has recently seen a surge in popularity, generally credited to its outstanding software modularity and standard code library (Muller et al., 2015). Similarly, EyeLoop’s internal algorithm is modular: Experiments are built by combining modules, native or otherwise, with the *Core* engine (Figure 1).

Internally, EyeLoop consists of two domains: An engine and an array of external modules. The engine detects the pupil and corneal reflections, while the modules essentially import or extract data to and from the system, respectively. *Extractor* modules are thus commonly used for data acquisition or experimental schemes, such as closed loops. In turn, *Importer* modules import video sequences to the system, such as from a camera feed.

The graphical user interface is a module as well, enabling users to adapt the system to any application, such as optogenetic experiments or educational schemes. Generally, EyeLoop’s high modularity greatly improves its compatibility across software versions, hardware specifications, and camera types.

The engine processes each frame of the input video sequentially (Figure 1A_1_): Each video frame is received by the EyeLoop engine as it is externally triggered, for instance, by an automatic video feed (e.g., using a consumer-grade web-camera), or manually (e.g., using research-grade cameras by TTL or BNC). This enables users to synchronize EyeLoop to external behavioral or electrophysiological systems.

After receiving the video frame, it is binarized, filtered, and smoothed by a Gaussian process (Figure 1A_2_,_3_). While EyeLoop provides an estimated initial set of parameters for video frame thresholding and filtering based on the pixel distribution, users are typically required to optimize the parameter set to obtain ideal processing conditions, e.g., high contrasts and smooth contours. This is done using key-commands, see *Default graphical user interface* in Supplementary Materials.

Next, the coordinates of the corneal light reflections and the pupil are selected manually by user input (Figure 1A_2–4_). Based on this initial position estimate, EyeLoop detects the contours of the pupil and corneal reflections based on a novel variation on Sakatani and Isa (2004) iterative walk-out method. Our vectorized algorithm extracts the four cardinal axes and *x* diagonals from the image matrix (where *x* can be any integer). Specifically, the diagonals are given by the variable step-sizes *m* and *n* according to the definition *D* = *d*_*mi, nj*_. The cardinal axes and diagonals are mapped onto Boolean matrices, which are used to mask the thresholded video feed. This provides targeted “*array views*” of the video frame matrix that can be tested against a binary condition to detect edges. Since the pupil consists of white pixels in the thresholded transform (value = 1), the first occurrence of a black pixel (0) in the array view is returned as an edge position. This is achieved *via* the Python module *NumPy*:


diagonal_edge=numpy.argwhere(video[diagonal_mask]==0)

The detection of the pupil/corneal reflection contours are thus reduced to repeated matrix computations (extract view, test binary condition, return contour points, …), which can be distributed across multiple central processing unit (CPU) cores during runtime for advanced use-cases. In contrast to the conventional iterative method, our vectorized approach enables computational operations to run in well-optimized C-code, which greatly benefits its efficiency. Likewise, the vectorized method ports easily to efficient, low-level machine code, e.g., *via Numba*.

The walk-out algorithm generates a matrix of points along the ellipsoid contour that is subsequently filtered based on the distance of each point from the mean (Figure 1B). Specifically, EyeLoop computes the mean of the contour matrix, the difference of each point from this mean, and the standard deviation of the set of distances from the mean. Points that are located more than 1 standard deviation from the mean are discarded. Since the mean approximates the center of the pupil, filtering performance can be improved by increasing the number of data points (by varying the diagonal step size). In general, more data points offer better tracking accuracy at a slight cost to tracking speed. The data generated by EyeLoop for this article was based on 32 contours points, which is also the default setting. This number strikes a balance between speed and accuracy for video frame sizes of up to 300 × 300. At larger video frame sizes, the number of contour points should be elevated as well to account for a larger pupillary circumference in video coordinates.

The ellipsoid outlined by the contour points is next parameterized and modeled as either a general ellipsoid shape (suitable for off-axis recordings, cats, rodents, …) (Halır and Flusser, 1998; White et al., 2010; Hammel and Sullivan-Molina, 2019) or a perfect circle (on-axis recordings in human, non-human primates, rodents, …) (Kanatani and Rangarajan, 2011). Notably, in cases where visual obstructions are significant, e.g., eyelids, whiskers, and shadows, EyeLoop may benefit from the more restrictive fitting of a perfect circle. On the other hand, when the eye is captured significantly off-axis, the video distortion of the pupil might make the general ellipsoid fitting more suitable (Świrski et al., 2012). Thus, the choice of the fitting algorithm extends beyond the animal species (Banks et al., 2015), and should include considerations about the video conditions, especially the camera angle, as well (Świrski et al., 2012). We have used circular tracking for all of this article’s data, since the camera angle was on-axis (orthogonal), and pupil shapes were round (mouse, human, primate). Human and non-human primate data are available in Supplementary Videos 1, 2.

Finally, as the next video frame is received by the *Importer*, the pupil and corneal reflection positions are re-estimated based on the previous frame’s ellipsoid fit center. In cases where the position of the pupil/corneal reflection deviate excessively between frames, e.g., due to blinking, EyeLoop falls back on a robust, yet more computationally expensive ellipse-detection algorithm based on the Hough transform: The most probable ellipsoid is selected based on position, size, and pixel distribution. If no suitable ellipsoids are detected, e.g., due to the eye being closed, the frame is marked as a blink. When a suitable ellipsoid is detected, the pupil center is reset, and EyeLoop’s contour detection is applied again.

Together, this vectorized, mixed-algorithm approach enables EyeLoop to consistently run at speeds of more than 1,000 frames per second, operated solely by the CPU. By contrast, cutting-edge deep learning methods on the CPU currently peak at speeds near 50 frames per second (Mathis and Warren, 2018).

## Results

### EyeLoop vs. DeepLabCut

**FIGURE 2:**
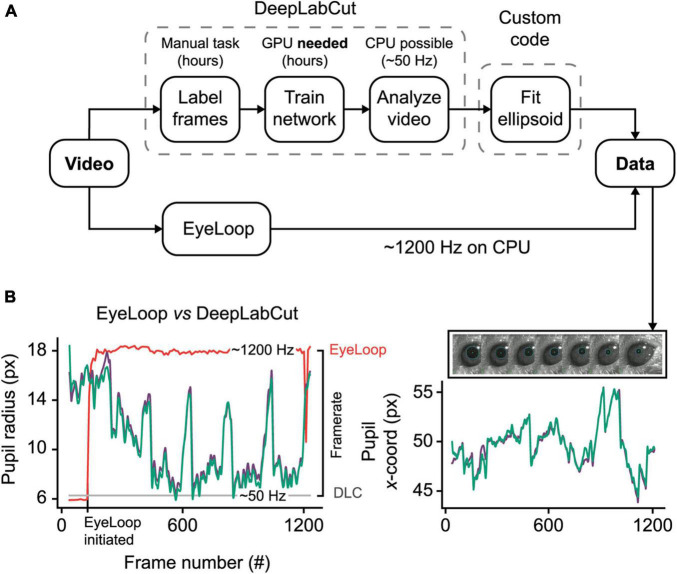
EyeLoop compared to DeepLabCut eye-tracking. **(A)** Schematic comparison. DeepLabCut requires several steps of setup before tracking can be initiated. Eye-tracking using DeepLabCut on the CPU is limited to ∼50 Hz. In contrast, EyeLoop requires minimal setup and runs at speeds greater than 1,000 Hz on the CPU. **(B)** Data comparison. EyeLoop and DeepLabCut produce similar data despite a significant gap in computational load. Green and purple lines are EyeLoop and DeepLabCut data, respectively. Red and gray lines are EyeLoop’s and DeepLabCut’s framerate, respectively.

DeepLabCut is a new deep neural network method for marker-less pose estimation (Nath et al., 2019), which is increasingly being applied to eye-tracking (Meyer et al., 2020). Due to its high accuracy and robustness, DeepLabCut presents an excellent eye-tracking reference for EyeLoop. The main disadvantage of DeepLabCut is its hardware intensiveness, requiring a dedicated processing unit for real-time operation (Mathis and Warren, 2018). Besides, the initial setup of DeepLabCut is time-consuming, generally spanning several hours of manual image labeling and subsequent neural network training. By contrast, EyeLoop operates at very high speeds on the general-purpose CPU with minimal initial setup needed (Figure 2).

To generate the reference dataset, we trained a DeepLabCut neural network to detect 8 points along the pupil periphery. We then fitted an ellipsoid to DeepLabCut’s data points, which were confirmed to have ideal eye-tracking accuracy by visual inspection. The comparison shown in Figure 2B and Supplementary Video 3, reveals a high similarity between DeepLabCut and EyeLoop’s eye-tracking data, both in terms of absolute coordinates (0.015 ± 0.518 px) and ellipsoid fitting (0.357 ± 0.438 px^2^). Generally, EyeLoop slightly underestimated the ellipsoid area compared to DeepLabCut. The reason for this is shown in Figure 1B: Since EyeLoop optimizes its detection of the contour by filtering its data points around occlusions, it will inherently tend to underestimate the true pupil outline. This underestimation can be minimized by increasing the number of data points. Notably, in the case presented here, EyeLoop uses 32 data points to extract the pupil contour – a good balance between speed and accuracy – while DeepLabCut suffices with 8 points. This difference in quantity is explained by DeepLabCut’s general robustness at detecting image features, specifically the true outer contour of the pupil, while ignoring false contours, e.g., eyelid overlap or reflections. Since EyeLoop is based on a more specific algorithm, it benefits from a higher quantity of markers to reduce artifacts from noise and obstructions.

Despite this operational difference, EyeLoop operates at processing speeds greater than 1,000 Hz on a consumer-grade CPU (Intel i7 8700K, single-core performance), which far exceeds the speeds currently achievable with DeepLabCut on the CPU (∼50 Hz, Intel Xeon E5-2603 v4, multi-core performance) and with a high-end GPU (200–500 Hz, GTX 1080 Ti), even when significantly downsampled (Mathis and Warren, 2018). High processing speeds are critical for several types of experiments, including closed loop experiments that require very fast feedback, and experiments examining delicate eye movements, such as micro-saccades (> 600 Hz), post-saccadic oscillations (> 500 Hz), and fixation (Juhola et al., 1985; Nyström et al., 2013). Moreover, high-frequency sampling provides a high signal-to-noise ratio, making statistical tests less laborious (Andersson et al., 2010). These findings altogether demonstrate that EyeLoop is a valuable alternative to DeepLabCut for high-speed eye-tracking. Yet, when speed is of no concern, or when the video material is of poor quality (e.g., contains frequent whisking, blinking), DeepLabCut may be a better choice for more robust eye-tracking performance.

### Open-Loop Experiment

**FIGURE 3:**
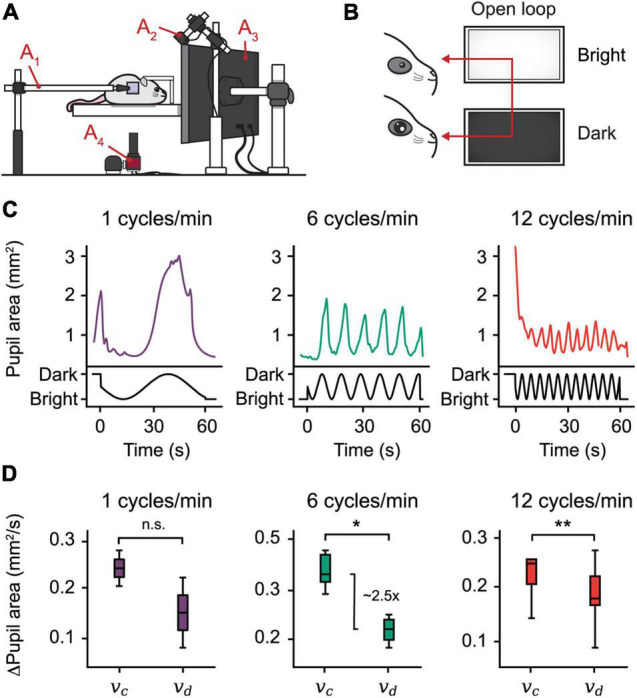
Open-loop experiments reveal pupillary reactivity dynamics in mice. **(A)** The setting used for eye-tracking in mice. A hot mirror is positioned beside the mouse and above the camera **(A_1–4_)**. A monitor displaying the visual stimulus is positioned facing the mouse **(A_3_)**, while a near-infrared light source is placed in the back **(A_2_)**. **(B)** Open-loop experiment. A sine function is mapped onto the brightness of a monitor, producing oscillations in the pupil area. **(C)** Plots from three open-loop experiments with frequencies 1, 6, and 12 cycles/min. **(D)** Constriction speed (v_*c*_) and dilation speed (v_*d*_) for each frequency calculated using the first derivative of the pupil area plots. The centerline is median, box limits are 25th and 75th percentiles, and whiskers show the minimum and maximum values. *P < 0.05, **P < 0.01, n.s., not significant, Wilcoxon signed-rank test.

To demonstrate the utility of EyeLoop in open-loop experiments, we designed an *Extractor* module that modulates the brightness of a monitor based on the phase of the sine wave function (Figure 3 and Supplementary Figure 1B). Using this design, we examined the pupillary reactivity to a light stimulus in awake mice.

More concretely, the size of the pupil is modulated by a special class of intrinsically photosensitive cells in the retina that projects to the upper midbrain and modulates pupil size in the *pupillary light reflex* (Lucas et al., 2003; Markwell et al., 2010). Accordingly, as the light dims, the pupil dilates to let more light through the iris onto the retina. Crucially, pupillary reactivity to light is a common parameter by which clinicians assess patient neurological status. For example, it has been demonstrated that abnormal pupillary light reactivity correlates with elevated intracranial pressures, possibly reflecting undiagnosed disease (Chen et al., 2011). Providing an accessible method to assess pupillary reactivity thus presents an attractive clinical use-case of EyeLoop.

To examine pupillary reactivity, we modulated the brightness of a PC monitor *via* three sine-wave frequencies ranging from 1 to 12 cycles/min; with increasing frequency, the monitor brightness cycled more rapidly through dim and bright settings. Using this setup, our findings confirm, first, that pupil size entrains to monitor brightness by inverse proportionality, a predictable consequence of the pupillary light reflex (Figure 3C). Second, using the pupil area’s first derivative, we found that the pupillary constriction speed dominates the speed of dilation in mice, which mirrors findings in humans (Figure 3D; Ellis, 1981). Taken together, these findings show that EyeLoop is well-suited to examine pupillary reactivity in living subjects.

### Optokinetic Reflex in Congenital Nystagmus Model vs. Wild-Type Mice

**FIGURE 4:**
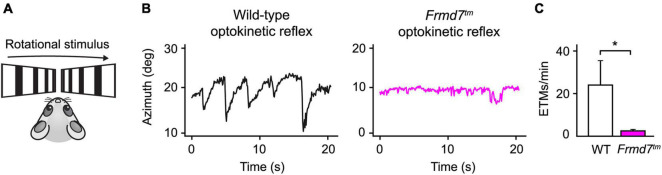
The horizontal optokinetic reflex is absent in Frmd7 knockout mice. **(A)** Rotational motion simulation using gratings drifting in parallel along the horizontal axis. **(B)** Eye movements evoked by the optokinetic reflex in wild-type and Frmd7™ mice in response to drifting grating stimulation. The azimuth represents the horizontal angular coordinate of the eye. **(C)** The optokinetic reflex was quantified as eye-tracking movements per minute (ETMs), computed by thresholding the first derivative of eye movements, as described by Cahill and Nathans (Cahill and Nathans, 2008). Error bars show standard deviation. *P < 0.05, Wilcoxon signed-rank test.

Often, neurological disorders, such as an undiagnosed brain hemorrhage or Horner’s syndrome, generate distinct abnormalities of the eyes. Similarly, patients suffering from congenital nystagmus exhibit flickering eye movements due to a failing optokinetic reflex. Detecting such neuropathological manifestations is crucial for early clinical diagnosis and biomedical research protocols. To show how EyeLoop may be applied to these ends, we confirmed previous findings showing that *Frmd7* hypomorphic mice lack the horizontal optokinetic reflex; similar to *Frmd7*-mutated congenital nystagmus patients (Yonehara et al., 2016). More concretely, we compared the optokinetic reflex of wild-type and *Frmd7* knockout mice, in which exon 4 of *Frmd7* was deleted from the genome, thus aiming to extend phenotypic reports on the hypomorphic genotype (Yonehara et al., 2016). To evoke the optokinetic reflex, we simulated a rotational motion using a bilateral drifting grating stimulus (Figure 4A). As expected, for wild-type mice, the optokinetic reflex was faithfully evoked (Figure 4B), whereas the reflex was absent in *Frmd7* knockout mice (Figure 4C). EyeLoop thus successfully verified the *Frmd7* hypomorphic phenotype in the complete knockout strain.

## Discussion

Conventional systems for eye tracking are typically tailored to large eyes, such as in human patients or non-human primates. For this reason, these systems often perform less accurately in rodents, where whiskers and eyelids tend to occlude the pupil. EyeLoop filters out occlusions by generating a highly detailed pupil marking. Thus, EyeLoop presents an attractive system for rodent biomedical research to investigate disease models, such as congenital nystagmus (Figure 4). Similarly, we recently applied EyeLoop in our lab to monitor the optokinetic reflex and investigate optic flow computations in visual cortices (Rasmussen et al., 2021).

EyeLoop fills an important gap as a tool to investigate the role of the eyes in brain processes. Sensory integration is complex, and often the eyes play an instrumental role in its orchestration. Eye-tracking during sensory exploration carries enormous information on how the senses are used by the brain: For example, during fast whole-body rotation, the eyes act to stabilize the gaze *via* the vestibulo-ocular reflex by integrating both vestibular and visual signals (Fetter, 2007). Yet, despite the known complexities of sensory computations, visual experiments are usually aimed at strictly monitoring the eyes (Meyer et al., 2020) or at applying one-sided stimuli in open loops (de Jeu and De Zeeuw, 2012). EyeLoop integrates the eyes as experimental items, providing pupil parameters for online processing and analysis. EyeLoop’s very high speed enables rapid experimental loops that are crucial for investigating visual and neural dynamics. Indeed, fine eye movements, such as post-saccadic oscillations and micro-saccades, are only discernible at high sampling frequencies (preferably greater than 1,000 Hz) (Juhola et al., 1985; Nyström et al., 2013), which is currently offered by no other open-source software than EyeLoop. Similarly, investigating the dynamics of neural learning and plasticity requires a very precise timing of learning cues, e.g., based on pupil size and arousal state (McGinley et al., 2015; Costa and Rudebeck, 2016; Wang et al., 2018; de Gee et al., 2020). EyeLoop’s seamless integration of experimental protocols, *via* its *Extractor* class, enables researchers to design loops that iterate at high speeds (> 1,000 Hz) to reveal causal relations of neural dynamics. Future experiments could thus apply EyeLoop to silence or stimulate specific neuronal populations *via* optogenetics to investigate the causality between neuronal activity and the endogenous parameters by which the nervous system operates (Grosenick et al., 2015).

## Limitations of the Study

The accuracy of EyeLoop hinges on the quality of the video frames, so illumination and contrasts should be optimized to get the best results. Additionally, EyeLoop is vulnerable to frame-to-frame inconsistencies, such as after prolonged blinking. To counter this vulnerability, EyeLoop falls back on the Hough Transform in cases where its main algorithm fails. This enables EyeLoop to run on inexpensive hardware at very high speeds, yet at a cost on robustness compared to well-trained, deep learning-based approaches (Nath et al., 2019). Along the same vein, EyeLoop’s edge detection is vulnerable to visual obstructions that cannot be sufficiently filtered by thresholding and Gaussian mapping, such as dense whiskers and significant eyelid overlap. Deep-learning methods, however, are often limited to offline processing due to hardware-intensive operations. Despite these limitations, EyeLoop provides an attractive balance between speed, accuracy, and robustness, which enables high-speed closed-loop experiments by high-level programming.

## Data Availability Statement

The datasets presented in this study can be found in online repositories. The names of the repository/repositories and accession number(s) can be found below: https://github.com/simonarvin/eyeloop.

## Ethics Statement

The animal study was reviewed and approved by Danish National Animal Experiment.

## Author Contributions

SA and KY conceived, designed the project, interpreted the data, and wrote the manuscript. SA developed the software and analyzed the data. SA and RNR performed the experiments. All authors contributed to the article and approved the submitted version.

## Conflict of Interest

The authors declare that the research was conducted in the absence of any commercial or financial relationships that could be construed as a potential conflict of interest.

## Publisher’s Note

All claims expressed in this article are solely those of the authors and do not necessarily represent those of their affiliated organizations, or those of the publisher, the editors and the reviewers. Any product that may be evaluated in this article, or claim that may be made by its manufacturer, is not guaranteed or endorsed by the publisher.
