# Supplementary figures and images for: EyeLoop: An Open-Source System for High-Speed, Closed-Loop Eye-Tracking
Source: Front Cell Neurosci. 2021 Dec 9;15:779628. doi: 10.3389/fncel.2021.779628 (PMC8696164; doi:10.3389/fncel.2021.779628)

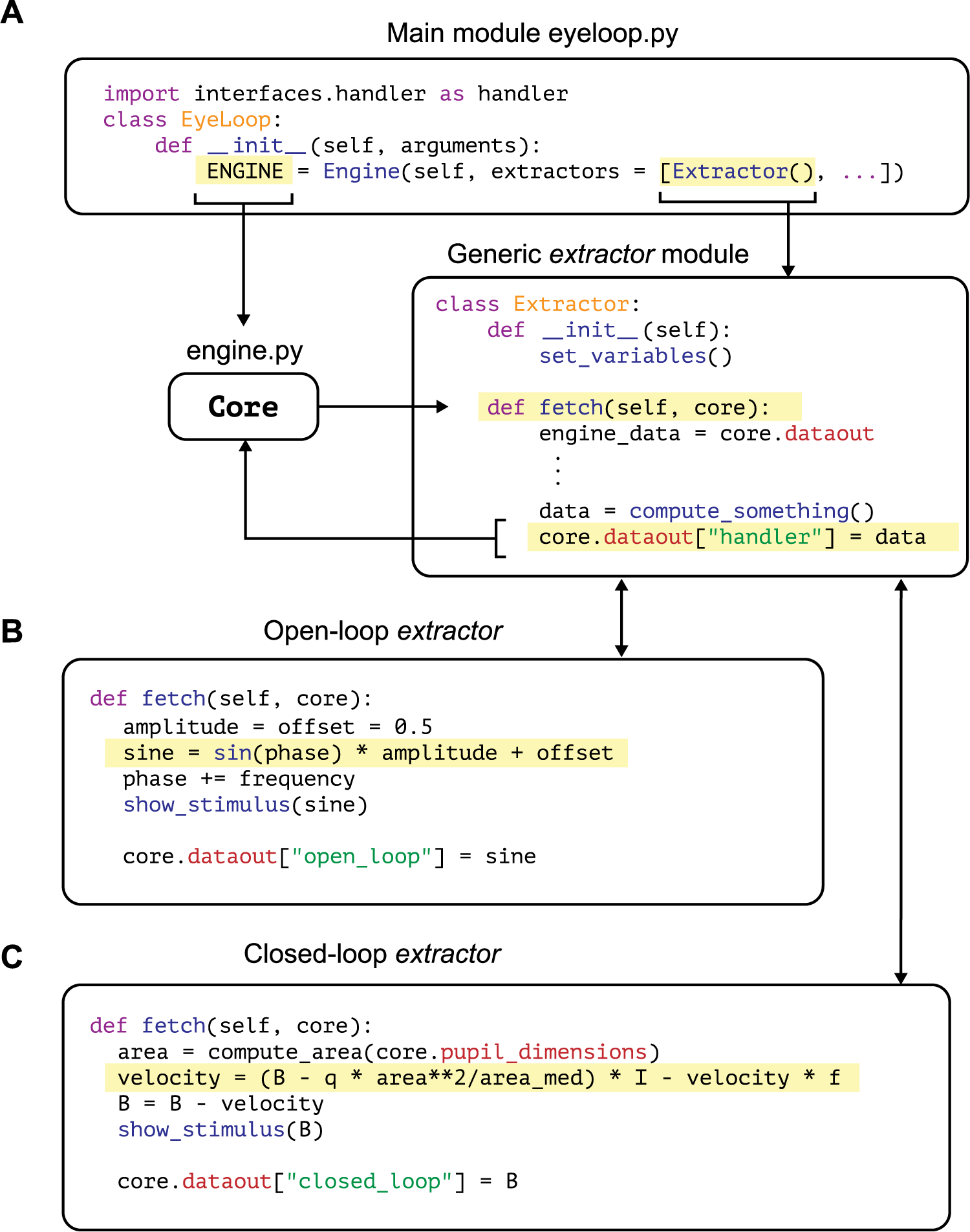

Supplement: Supplementary file 1 [file Image_1.TIF]
