# Supplementary material for: EyeLoop: An Open-Source System for High-Speed, Closed-Loop Eye-Tracking
Source: Front Cell Neurosci. 2021 Dec 9;15:779628. doi: 10.3389/fncel.2021.779628 (PMC8696164; doi:10.3389/fncel.2021.779628)
Supplement: Supplementary file 5 [file Data_Sheet_1.docx]

**Supplementary material**

**Getting started (technical guide)**

*Install*

EyeLoop is open-source software, enabling users to download and modify its source code freely. To download EyeLoop, go to its git repository to obtain a composite zip-file (<https://github.com/simonarvin/eyeloop>) or download it directly via git:

*git clone* https://github.com/simonarvin/eyeloop.git

To avoid interfering with other software systems, it is recommended to install and run EyeLoop within a virtual environment. For example, using Python’s standard virtual environment, *venv*:

*python -m venv venv*

The virtual environment is then activated according to the operating system in use. Next, to install EyeLoop, go to its download directory using command-line *cd*, then run:

*pip install .*

EyeLoop should now be successfully installed on the system or virtual environment. To update EyeLoop, simply repeat the steps above. Importantly, when modifying EyeLoop files, e.g., for experimental purposes, virtual environment users should do this specifically within their virtual environment directory; Otherwise, changes will fail to take effect. Alternatively, EyeLoop must be reinstalled from the modified source code.

*Initiate*

To initiate EyeLoop, open the command-line terminal and run:

*eyeloop*

Image data is fed to the system either online (in real-time from a camera) or offline. To switch between input modes, use the argument tag “—*video*”. Specifically, to route the live feed from a video camera *n*, run:

*eyeloop* —*video* *n*

Where *n* = 0, 1, 2, etc., yet by default *n* = 0. If the video camera requires specialized software for image acquisition, choose an appropriate *Importer* module using the argument tag “—*importer*”. EyeLoop natively offers a default *opencv*-based *Importer* (suitable for most cameras) and a *Vimba*-based *Importer* (suitable for *AlliedVision* cameras). For example, to select the Vimba-based *Importer*, run:

*eyeloop* —*video* *n* —*importer vimba*

Alternatively, to import a prerecorded video sequence, simply pass the video file path or image directory via argument tag “—*video*”:

*eyeloop* —*video* *[file-path or image directory]*

Before the trial starts, users may also want to inspect the pupil type that is to be tracked; EyeLoop is, by default, tailored to rodent eye-tracking via a general ellipsoid fitting model. However, to switch to a circular model, suitable for primate eye-tracking, use the argument tag “—*model circular*”:

*eyeloop* —*model circular*

To track human eyes using an AlliedVision (Vimba-based) camera on port 1, for instance, run:

*eyeloop* —*video 1* —*importer vimba* —*model circular*

On the initial run, EyeLoop will calibrate its blink detection algorithm. Users are recommended to wait for the calibration to complete. On subsequent runs, the calibration file can be loaded from the data directory using the command line argument:

*eyeloop* —*blink [path to calibration-file]*

Finally, to list all argument tags, run the command:

*eyeloop —help*

Sample test data can be found in EyeLoop’s “*Playground*” repository, see <https://github.com/simonarvin/eyeloop_playground>

*Default graphical user interface*

EyeLoop’s graphical workspace is customizable. To offer EyeLoop to a broad audience, we designed a minimal default user interface called *minimum-gui*. *Minimum-gui* contains a graphical overview of both the raw and processed video feeds and a tutorial bottom panel. To start tracking, the user selects the corneal reflections by hovering with the computer cursor and key-pressing *2*, *3,* or *4*. This initiates the tracking process, which is rendered in the preview panel. The user may adjust binarization (keys *W or S*) and gaussian (keys *E or D*) parameters to improve detection. When the corneal reflections have been selected, the user hovers over the pupil and keypresses *1*. The user again may adjust binarization (keys *R* or *F*) and gaussian parameters (keys *T* or *G*) for optimal detection. These settings are automatically saved, and can be reloaded by EyeLoop using the command line argument:

*eyeloop* —*params [path to params-file]*

Parameter files are by default saved in the eye-tracking session’s data directory, e.g., “*…/trial_20211104-142722” (*see *“Retrieve data offline”* below*)*. Parameters for sample videos are provided in EyeLoop’s supplemental *“playground”* repository, see <https://github.com/simonarvin/eyeloop_playground>.

For some experiments, the user may want to rotate the video feed to align it with the real-world axes. Thus, to rotate the video feed counterclockwise or clockwise, use keys *O* or *P*, respectively. Alternatively, eye-tracking data can be rotated post hoc by an *angular transformation* module using basic matrix operations.

Finally, to initiate a high-speed eye-tracking session, key-press *Z*, then confirm by *Y* (or return by *N*). **Note:** During high-speed tracking, the video display is updated at time intervals that may not reflect the actual processing speed. While it is recommended to leave the update interval to default, it can be adjusted using:

*eyeloop* —*fps [seconds]*

*Build an experiment*

Users can build experiments in EyeLoop by linking *Extractor* modules to the *Core* engine. *Extractors* contain an *instantiating* function called at startup, and a *fetch* function called once every time-step. The *Extractor* module has access to all eye-tracking data in real-time via the *Core* pointer (**Figure 2**). By default, EyeLoop initiates a base set of *Extractors*, namely a framerate counter and a data acquisition tool. However, users can append any number of custom *Extractors* to the base. As an example, we here briefly describe how to set up an *Extractor* to continuously read and print the pupil size.

*Extractors* are essentially Python class elements with unique identifiers. We thus first create a new Python file, e.g., “*test_extractor.py*”, and add a class entry “*Test_extractor*”. We then add the basic *Extractor* class functions, *__init__* and *fetch*:

*class Test_extractor:*

*def __init__(self):*

*print(“Test extractor initiated.”)*

*def fetch(self, core):*

…

Using the *fetch* function, we retrieve the width and height of the pupil from the *Core* engine’s *dataout* dictionary:

*def fetch(self, core):*

*width, height = core.dataout["pupil"][0]*

Next, we calculate the pupil area and print the result:

*area = width * height * pi*

*print(“pupil area: ”, area)*

We may also add the pupil area to the *Core* engine’s dictionary data output *core.dataout*, so it is included automatically by the data acquisition *Extractor*:

*core.dataout[“pupil_area”] = area*

Finally, to make sure our test *Extractor* is initiated as EyeLoop starts up, we add it to the *final* *Extractor* list. We do this by defining an *extractors_add* array within our *Extractor* file:

*extractors_add = [Test_extractor()]*

*extractors_add* and *extractors_base* are combined in EyeLoop’s main file:

*extractors = extractors_add + extractors_base*

*config.engine.load_extractors(extractors)*

We may now finally test our *extractor* by running EyeLoop with argument tag “—*extractors*”:

*eyeloop* —*extractors path/to/test_extractor.py*

Alternatively, passing —*extractors p* EyeLoop launches a file dialog window.

*eyeloop* —*extractors p*

More advanced *Extractors* are essentially designed along the same vein (**Figure S1**). Similarly, multiple *Extractors* may be added by using a composite Python script, e.g.:

*from extractor1 import extractor1*

*from extractor2 import extractor2*

*extractors_add = [extractor1(), extractor2()]*

*Retrieve data offline*

All EyeLoop’s data are stored in the "*attribute—value*”-based JSON-format. Each trial generates a unique data directory identifiable by its timestamp, e.g.,

*…/trial_20211104-142722*

Inside this directory, EyeLoop’s data is stored in *datalog.json.* To load each frame of the data, open the JSON-file, and read it line by line:

*import json*

*data_path = r”trial_X/datalog.json”*

*with open(data_path, “r”) as file:*

*for data_entry in file.readlines():*

*print(data_entry)*

Each data entry contains a time-attribute, retrievable by:

*data_entry[“time”]*

Similarly, the pupil and corneal reflection data are retrievable by:

*data_entry[“pupil”]*

*data_entry[“cr1”]*

*data_entry[“cr2”]*

Each ellipsoid entry is an array containing the following elements:

*((center_x, center_y), radius1, radius2, angle)*

For instance, to retrieve the position of the pupil:

*pupil_position = data_entry[“pupil”][0]*

Basic functions to retrieve data are provided in the EyeLoop-package by default, see “*utilities/parser.py*”.
